# Supplementary figures and images for: DeepCINAC: A Deep-Learning-Based Python Toolbox for Inferring Calcium Imaging Neuronal Activity Based on Movie Visualization
Source: eNeuro. 2020 Aug 12;7(4):ENEURO.0038-20.2020. doi: 10.1523/ENEURO.0038-20.2020 (PMC7438055; doi:10.1523/ENEURO.0038-20.2020)

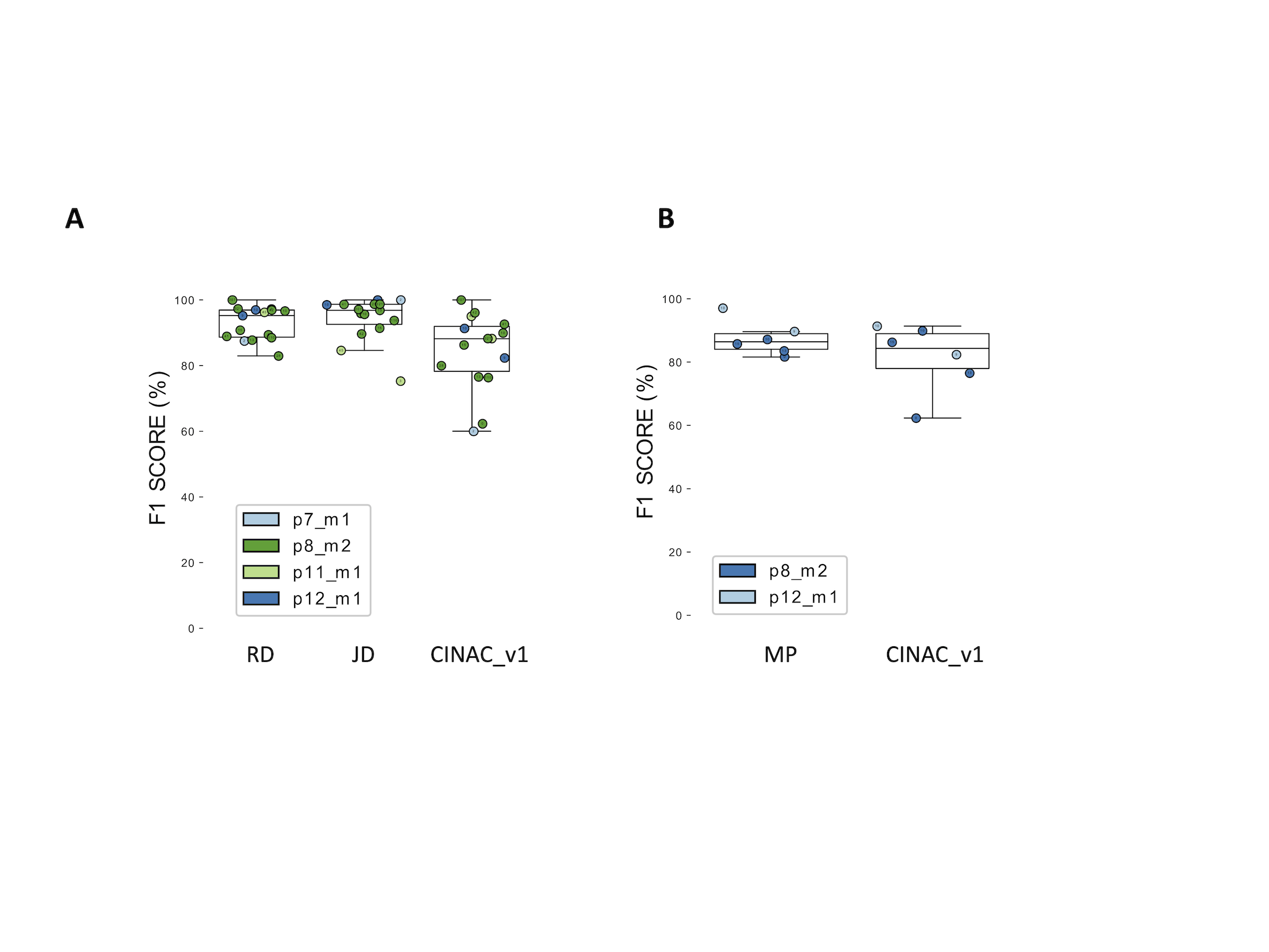

Supplement: Extended Data Figure 7-1 — Comparison of CINAC performance to human experts. A, Boxplot displaying F1 score of two human experts (R.F.D. and J.D.) and CINAC_v1. Here are shown 15 cells annotate by both experts. B, Boxplot displaying F1 score of one human expert (M.A.P.) and CINAC_v1. Here are shown six cells annotated by M.A.P. Each colored dot represents a cell, the number inside indicates the cell’s id and each color represents a session as identified in the legend. CINAC_v1 is a classifier trained on data from the Hippo-dvt dataset (Table 1; Extended Data Table 1-1). Download Figure 7-1, TIF file. [file enu-eN-OTM-0038-20-s03.tif]

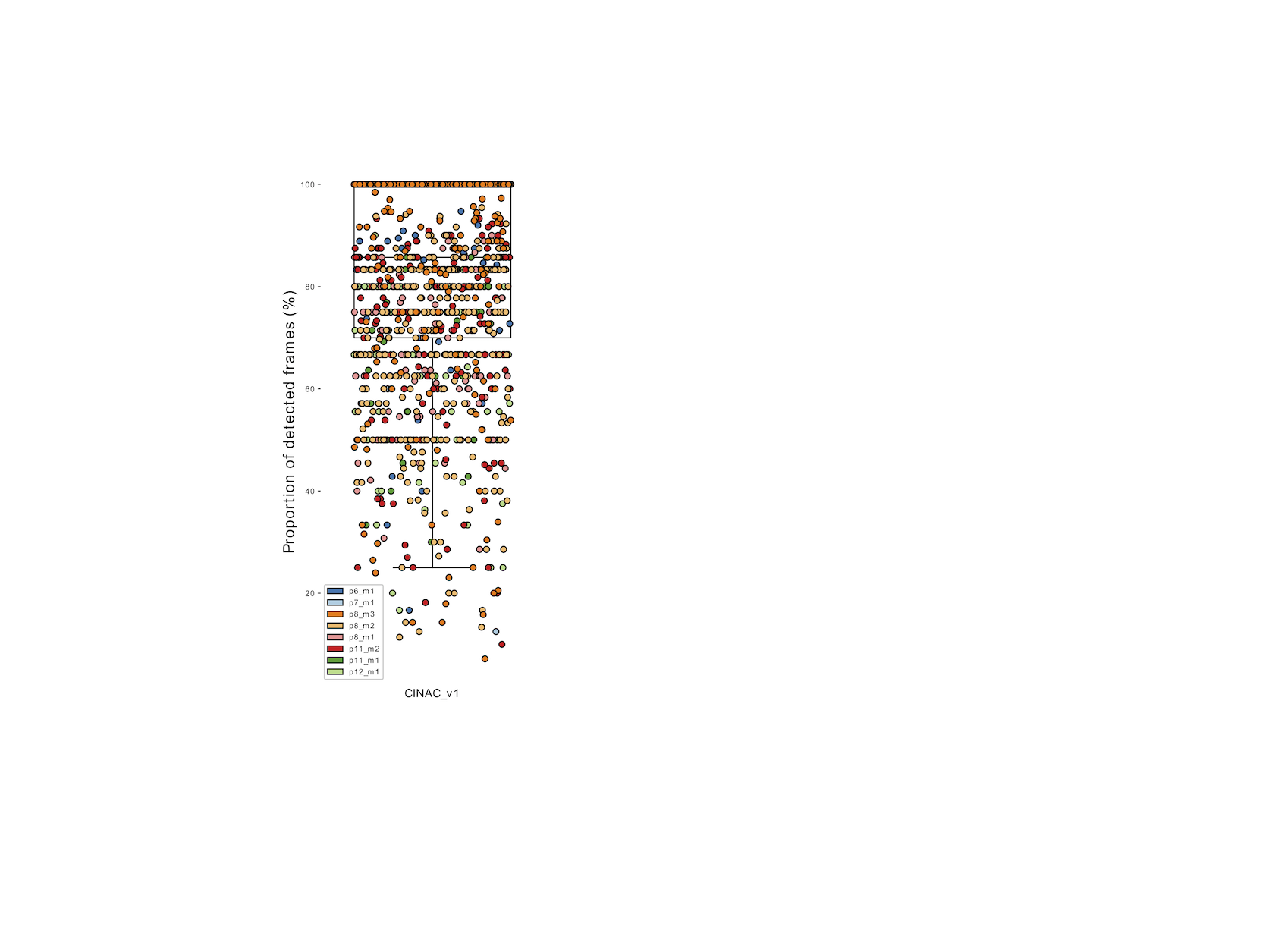

Supplement: Extended Data Figure 7-2 — Onset to peak detection of calcium transient. Boxplot showing the proportion of frames predicted as active during the transient rise time. CINAC_v1 is a classifier trained on data from the Hippo-dvt dataset (Table 1; Extended Data Table 1-1). Each colored dot represents a transient and each color represents a session as identified in the legend. Download Figure 7-2, TIF file. [file enu-eN-OTM-0038-20-s04.tif]

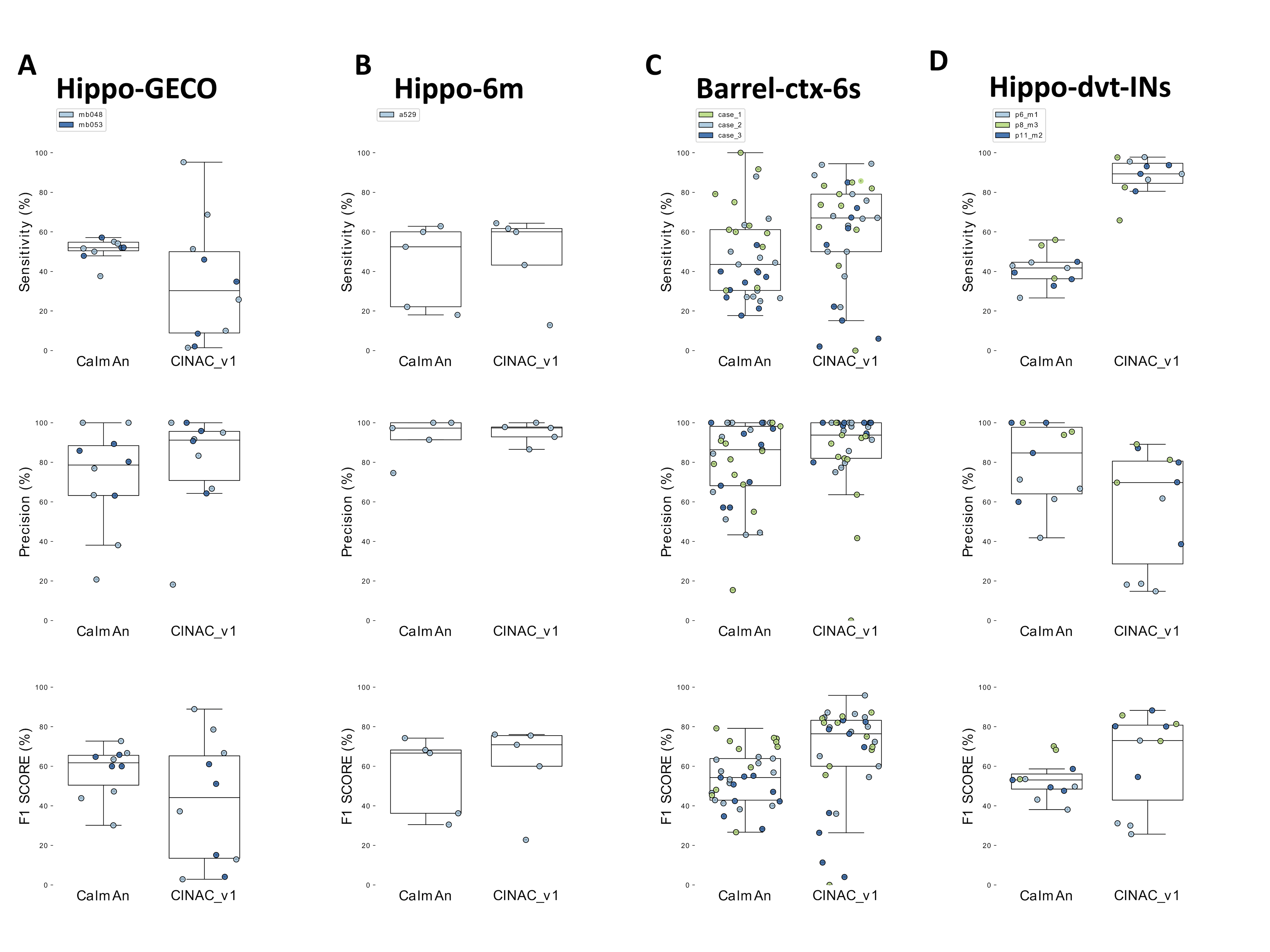

Supplement: Extended Data Figure 8-1 — Comparison of CaImAn and CINAC_v1 performances on various dataset. A, Boxplot displaying the sensitivity (top panel), precision (middle panel), and F1 score (bottom panel) for Hippo-GECO dataset. For each panel, we evaluated CaImAn performance as well as CINAC_v1. B, Boxplot displaying the sensitivity (top panel), precision (middle panel), and F1 score (bottom panel) for Hippo-6m dataset. C, Boxplot displaying the sensitivity (top panel), precision (middle panel), and F1 score (bottom panel) for Barrel-ctx-6s. D, Boxplot displaying the sensitivity (top panel), precision (middle panel), and F1 score (bottom panel) for Hippo-dvt-INs dataset. Each colored dot represents a cell, the number inside indicates the cell’s id and each color represents a session as identified in the legend. CINAC_v1 is a classifier trained on data from the Hippo-dvt dataset (Table 1; Extended Data Table 1-1). Download Figure 8-1, TIF file. [file enu-eN-OTM-0038-20-s05.tif]

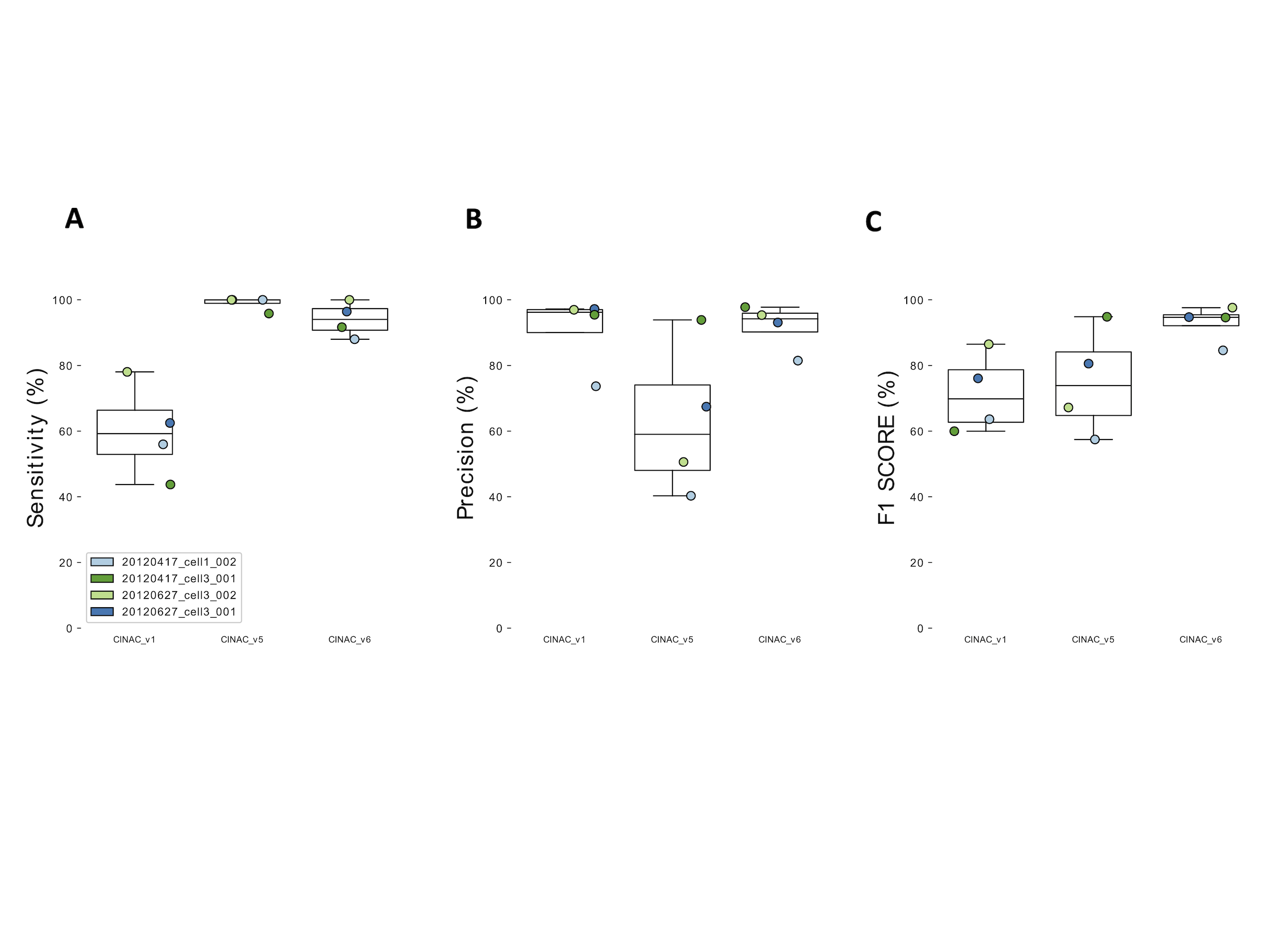

Supplement: Extended Data Figure 8-2 — Use of DeepCINAC classifiers to optimize performances on Visual-ctx-6s dataset. A, Boxplots showing sensitivity for CINAC_v1, CINAC_v5 and CINAC_v6 evaluated against the known ground truth of four cells from the GENIE project. B, Boxplots showing precision for CINAC_v1, CINAC_v5, and CINAC_v6 evaluated against the known ground truth of four cells from the GENIE project. C, Boxplots showing F1 score for CINAC_v1, CINAC_v5, and CINAC_v6 evaluated against the known ground truth of four cells from the GENIE project. CINAV_v1 is a classifier trained on data from the Hippo-dvt dataset, CINAC_v5 is a classifier trained on data from Visual-ctx-6s dataset, CINAC_v6 is a classifier trained on data from Visual-ctx-6s dataset and four cells from the Hippo-dvt dataset (Table 1; Extended Data Table 1-1). Each colored dot represents a cell. Cell labels in the legend correspond to session identifiers from the dataset. Download Figure 8-2, TIF file. [file enu-eN-OTM-0038-20-s06.tif]

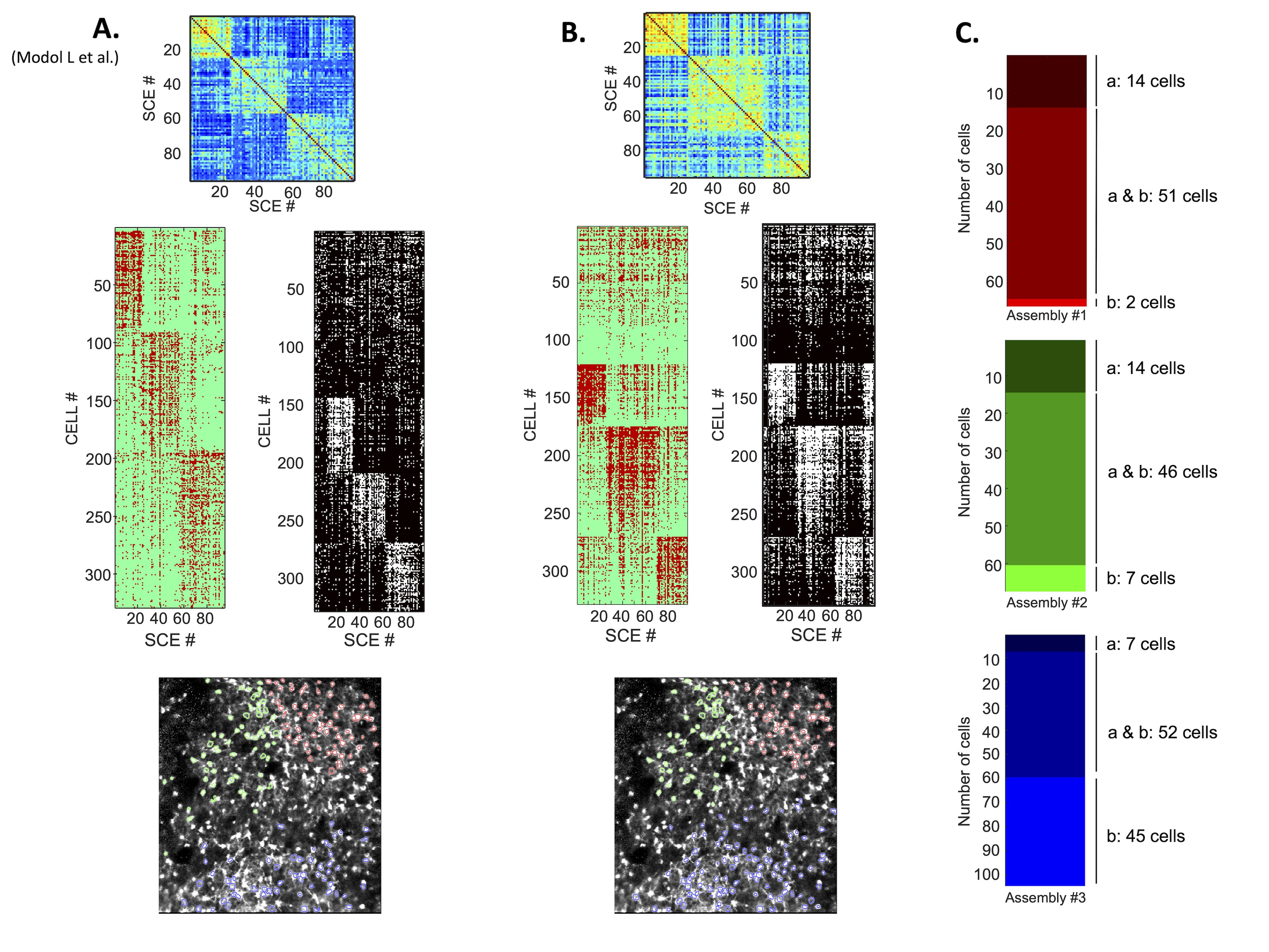

Supplement: Extended Data Figure 8-3 — Cell assemblies detection and organization using CaImAn and CINAC_v1 on published data. A, B, The top panel represents the clustered covariance matrix of synchronous calcium events (SCE). The middle panel represents neurons active in SCE organized by cluster (cell assembly). The bottom panel represents the cell’s map, each color represents a cell assembly. A, Cell assemblies detection results using CaImAn. B, Cell assemblies detection results using CINAC_v1. C, Individual cells composing assemblies in each method. an a represents the number of neurons detected by Modol et al. (2020), using CaImAn; an b represents the number of neurons detected using CINAC_v1. Each color represents a cell assembly, color coded as in the maps. Download Figure 8-3, TIF file. [file enu-eN-OTM-0038-20-s07.tif]
